# HIV-1 drug resistance mutations emerging on darunavir therapy in PI-naive and -experienced patients in the UK

**DOI:** 10.1093/jac/dkw343

**Published:** 2016-09-28

**Authors:** Kate El Bouzidi, Ellen White, Jean L. Mbisa, Caroline A. Sabin, Andrew N. Phillips, Nicola Mackie, Anton L. Pozniak, Anna Tostevin, Deenan Pillay, David T. Dunn

**Affiliations:** 1 Research Department of Infection and Population Health, University College London, London, UK; 2 Research Department of Infection, Division of Infection and Immunity, University College London, London, UK; 3 MRC Clinical Trials Unit at UCL, London, UK; 4 Virus Reference Department, Centre of Infections, Public Health England, London, UK; 5 Department of HIV Medicine, Imperial College Healthcare NHS Trust, London, UK; 6 Department of Medicine, Chelsea and Westminster Hospital NHS Foundation Trust, London, UK; 7 Wellcome Trust Africa Centre for Health and Population Sciences, University of KwaZulu Natal, Mtubatuba, South Africa

## Abstract

**Background:**

Darunavir is considered to have a high genetic barrier to resistance. Most darunavir-associated drug resistance mutations (DRMs) have been identified through correlation of baseline genotype with virological response in clinical trials. However, there is little information on DRMs that are directly selected by darunavir in clinical settings.

**Objectives:**

We examined darunavir DRMs emerging in clinical practice in the UK.

**Patients and methods:**

Baseline and post-exposure protease genotypes were compared for individuals in the UK Collaborative HIV Cohort Study who had received darunavir; analyses were stratified for PI history. A selection analysis was used to compare the evolution of subtype B proteases in darunavir recipients and matched PI-naive controls.

**Results:**

Of 6918 people who had received darunavir, 386 had resistance tests pre- and post-exposure. Overall, 2.8% (11/386) of these participants developed emergent darunavir DRMs. The prevalence of baseline DRMs was 1.0% (2/198) among PI-naive participants and 13.8% (26/188) among PI-experienced participants. Emergent DRMs developed in 2.0% of the PI-naive group (4 mutations) and 3.7% of the PI-experienced group (12 mutations). Codon 77 was positively selected in the PI-naive darunavir cases, but not in the control group.

**Conclusions:**

Our findings suggest that although emergent darunavir resistance is rare, it may be more common among PI-experienced patients than those who are PI-naive. Further investigation is required to explore whether codon 77 is a novel site involved in darunavir susceptibility.

## Introduction

Darunavir is a preferred antiretroviral agent in several HIV treatment guidelines for therapy-naive and experienced patients.^1–3^ This second-generation PI is generally well tolerated and is perceived to have a high genetic barrier to resistance. Darunavir-associated drug resistance mutations (DRMs) have been largely identified by analyses that examined the correlation between baseline genotype and virological response. However, there is less information on DRMs that are directly selected by darunavir in clinical settings.

Darunavir is considered less likely to cause clinically significant resistance than most PIs as it requires the HIV-1 protease gene to mutate several times to produce a corresponding reduction in phenotypic drug susceptibility.^4^ Eleven darunavir-associated DRMs are recognized by the International Antiviral Society (IAS)-USA.^5^ These occur at 10 protease positions and include six major mutations (shown in bold) and five minor amino acid substitutions: V11I, V32I, L33F, **I47V, I50V, I54L, I54M**, T74P, **L76V, I84V** and L89V. Darunavir DRMs were inferred from the POWER studies, clinical trials that established the efficacy of this agent in treatment-experienced patients, including those with baseline PI resistance.^6,7^ In the POWER studies, around half of participants that had baseline viruses with between zero and two DRMs achieved virological suppression at week 48, but this fell to 26% when three or more mutations were present. Two subsequent randomized controlled trials, TITAN and ARTEMIS, demonstrated the non-inferiority of darunavir compared with lopinavir in treatment-experienced and treatment-naive patients, respectively.^8,9^ The TITAN trial also showed a worse outcome in those with at least three pre-existing darunavir DRMs, as in this subgroup only 60% achieved viral suppression, compared with 90% overall.^8^ Observational data have been used to create genotypic resistance interpretation tools to predict the response to darunavir therapy based on the presence of specific baseline protease mutations. A study of 880 patients drawn from large European databases identified five baseline mutations that were associated with a reduced 8 week virological response to a darunavir-containing salvage regimen (L10F, V11L, I54M, T74P and V82I) and six mutations that were associated with an improved response (K20T, E34D, I64L, V82A, I85V and I93L).^10^

There are no studies to our knowledge of the emergence of darunavir resistance in clinical practice settings in the UK. The UK HIV Drug Resistance Database (UKHDRD) is a repository of genotypic resistance tests performed as part of routine clinical care, and currently contains over 100 000 HIV *pol* sequences.^11^ These are linked to demographic and clinical information provided by the UK Collaborative HIV Cohort (UK CHIC) study, which merges data from some of the largest HIV clinics in the UK.^12^ Over 6000 UK CHIC study participants have received darunavir since its introduction in 2007. This large national cohort with longitudinal viral genetic data presents an ideal opportunity to determine the DRMs that emerge during therapy in clinical practice. We aimed to identify emergent mutations by comparing the HIV-1 protease sequences obtained from individuals before and after darunavir exposure. We used a positive selection analysis approach that compared non-synonymous and synonymous mutations across the protease gene to identify codons not previously implicated in darunavir resistance.

## Patients and methods

UK CHIC participants (all aged over 16 years) were eligible for the study if they had received at least 30 days of a darunavir-containing regimen and had both a ‘baseline’ (defined as any time prior to darunavir exposure) and ‘post-exposure’ genotypic resistance test result (obtained either during darunavir therapy or within 30 days of stopping this agent). Participants were excluded if they had received another PI for ≥90 days between the baseline and post-exposure tests, to avoid attributing the effect of other agents to darunavir. A 90 day period was allowed to enable a resistance test result to be obtained and acted upon prior to switching to darunavir from another PI. Only genotypes with a complete protease sequence were considered. If more than one baseline genotypic test had been performed, the one closest to the start of darunavir was used. If more than one post-exposure test had been performed, the results were combined and therefore reflect cumulative resistance. All ART regimens that included darunavir were considered. Information on the dosing frequency (once or twice daily) was not recorded. The prevalence at baseline of darunavir DRMs, defined according to the IAS-USA 2015 list,^5^ was assessed from the baseline protease sequences. Emergent DRMs were identified by comparing the baseline and post-exposure sequences for each individual. Viral subtypes were determined by analysing the *pol* sequence with the REGA HIV subtyping tool version 3.^13,14^ All analyses were stratified by history of exposure to other PIs prior to initiating darunavir. Statistical analyses were performed with Stata/IC 13.1 software (StataCorp LP, College Station, TX, USA).

Selection pressure was examined by estimating non-synonymous (*dN*) and synonymous (*dS*) substitution rates during darunavir therapy using the HyPhy software package available on Datamonkey, a web-based interface.^15,16^ The *dN*:*dS* ratio was calculated for amino acid sites 5–99 of the protease gene and positive selection was inferred if *dN* > *dS*. The analysis was restricted to those with subtype B infection to avoid introducing bias from inter-subtype variability in WT amino acids and polymorphic loci. A case control approach was used for the positive selection analysis. Cases met the study eligibility criteria above and additionally had not been exposed to any other PIs prior to initiating darunavir. To distinguish the effects of darunavir therapy selection pressure from the natural evolution of the protease gene over time, we selected controls who met the same inclusion criteria, but who were PI-naive and had initiated an NNRTI-based ART regimen. For each case, two controls were randomly chosen, matched by calendar year of initiation of either darunavir or NNRTI. The selection pressure analysis was performed using three different codon-based algorithms: fixed effects likelihood (FEL), single likelihood ancestor counting (SLAC) and fast unconstrained Bayesian approximation (FUBAR) using a cut-off *P* value <0.05 for FEL and SLAC, and a posterior probability >0.95 for FUBAR.^17,18^ A starting phylogenetic tree was supplied for each analysis that was inferred by maximum-likelihood method in FastTree using a general time reversible nucleotide model of substitution.^19^ A single breakpoint recombination tree was used if recombination was detected. Sites were considered to be positively selected if this was confirmed by at least two of the three algorithms.

## Results

**Figure 1.**
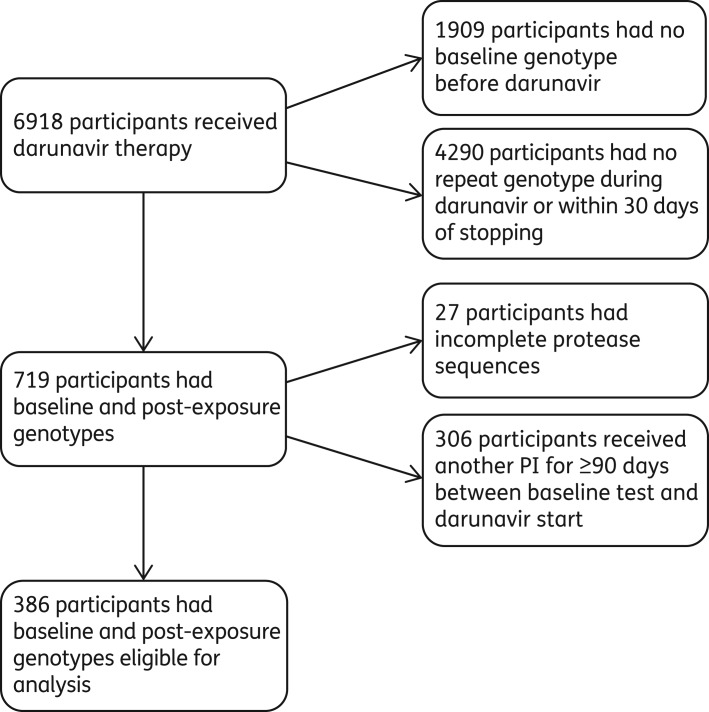
Flow chart: study participant selection.

A total of 6918 UK CHIC participants had received darunavir up to the end of 2012, of whom 386 met the inclusion criteria (Figure 1). Reasons for exclusion were lack of baseline and post-exposure resistance tests with complete protease sequences and receipt of another PI for ≥90 days following the baseline test. The characteristics of the study participants are shown in Table 1. There was a preponderance of men, with sex between men accounting for around half of the known modes of HIV acquisition. The majority of participants were viraemic at darunavir initiation. Of these 386 participants, 198 (51%) were PI-naive and 188 (49%) had a history of PI use prior to darunavir. In PI-experienced participants the most common previous PIs were lopinavir (135 participants, 72%), atazanavir (93 participants, 49%) and saquinavir (68 participants, 36%). One hundred and forty-eight (79%) PI-experienced participants were in receipt of ART at the time of the baseline resistance test compared with 54 (27%) PI-naive participants.

**Table 1.** Participant characteristics

	All participants (*n *= 386)	PI-naive (*n *= 198)	PI-experienced (*n *= 188)
Male sex, *n* (%)	284 (74)	155 (78)	129 (69)
Age (years), median (IQR)	42 (36–47)	41 (35–48)	42 (37–47)
Ethnicity, *n* (%)			
white	208 (55)	108 (56)	100 (54)
black	137 (36)	67 (35)	70 (38)
other	34 (9)	19 (10)	15 (8)
Mode of transmission, *n* (%)			
MSM	185 (51)	95 (52)	90 (50)
heterosexual	154 (43)	75 (41)	79 (44)
injection drug use	10 (3)	3 (2)	7 (4)
other	12 (3)	8 (4)	4 (2)
Subtype, *n* (%)			
A	37 (10)	17 (9)	20 (11)
B	208 (54)	108 (55)	100 (53)
C	67 (17)	37 (19)	30 (16)
CRF02_AG	32 (8)	16 (8)	16 (9)
other	42 (11)	20 (10)	22 (12)
Calendar year of darunavir start, median (IQR)	2009 (2008–10)	2010 (2009–11)	2009 (2008–10)
CD4+ cell count at darunavir start (cells/mm^3^), median (IQR)	242 (90–460)	248 (90–453)	235 (88–460)
Viral suppression at darunavir start, *n* (%)	28 (8)	18 (10)	10 (5)
HIV RNA at darunavir start (log_10_ copies/mL), median (IQR)	4.3 (2.9–5.2)	4.7 (2.8–5.5)	4.1 (2.9–4.9)
Time from baseline test to darunavir start (days), median (IQR)	51 (22–133)	71 (21–460)	43 (24–69)
Time from darunavir start to post-exposure test (days), median (IQR)	211 (106–441)	168 (68–370)	270 (133–500)
Viral suppression, RNA ≤50 copies/mL, occurred prior to post-exposure test, *n* (%)	171 (48)	79 (44)	92 (51)
Log_10_ drop in RNA if no viral suppression (log_10_ copies/mL), median (IQR)	0.3 (−0.2–1.8)	0.5 (−0.2–2.2)	0.3 (−0.2–1.1)
HIV RNA at post-exposure test (log_10_ copies/mL), median (IQR)	3.3 (2.4–4.5)	3.2 (2.3–4.6)	3.4 (2.6–4.5)

Missing data: ethnicity (4 PI-naive, 3 PI-experienced); mode of transmission (17, 8); CD4+ cell count (23, 12); HIV RNA at start (22, 6); RNA measurement between darunavir and post-exposure test (20, 7); RNA at post-exposure test (19, 8).

Overall, 11 of the 386 participants (2.8%) accumulated a total of 16 emergent darunavir DRMs during darunavir therapy: 4 × V11I, 4 × V32I, 2 × L33F, 2 × I54L, 3 × L76V and 1 × I84V (Table 2). The median time on darunavir until the post-exposure test with emergent DRMs was 5.8 months (IQR 2.1–10.0). The earliest emergent mutation was V32I, which was present after only 13 days of therapy, in a person who had a test showing WT protease sequence 1 month prior to starting darunavir. Baseline darunavir DRMs were present in the sequences of two (1.0%) of the PI-naive participants (T74P and L33F), compared with 26 (13.8%) PI-experienced participants, who had a total of 53 DRMs prior to darunavir exposure. Neither of the PI-naive participants with baseline mutations went on to develop emergent mutations in their post-exposure genotypes, but four (2.0%) PI-naive participants who did not have DRMs at baseline were found to have developed resistance on subsequent testing (2 × V11I, V32I, L76V). Among the PI-experienced group, seven people (3.7%) developed a total of 12 emergent DRMs, of whom five had also had baseline mutations. Not all baseline mutations were fixed as the dominant residues at those protease positions, and of the 55 DRMs present in baseline sequences, 12 were not detected in post-exposure tests.

**Table 2.** Participants with emergent IAS-USA darunavir mutations

Participants	IAS-USA darunavir mutations											Other IAS-USA PI mutations	Previous PI (*n*)	Subtype	Time to DRM^b^ (months)	ART with darunavir
	11I	32I	33F	47V	50V	54M	54L	74P	76V	84V	89V					
1	▪											36I, 60E, 69K, 89M, 93L	0	C	1.2	RAL
2	▪											10I, 20I, 36I, 69K, 89M	0	K	2.1	ABC/3TC
3		▪										10I, 36I, 62V, 77I, 93L	0	B	5.8	TDF/FTC
4									▪			10F, 20R, 36I, 46I, 54V, 69K, 71V, 82A, 89M, 93L	0	C	10.0	TDF/FTC
5	▪											62V, 63P, 77I, 93L	1	B	7.3	FTC/TDF
6			□							▪	□	10V, 36L, 48V, 54S, 58E, 62V, 63P, 71I, 73S, 82A, 90M	4^a^	B	9.2	T20/TDF
7		▪	□							□		10I, 20R, 36I, 48V, 54S, 62V, 63P, 64V, 71V, 82A, 93M	5^a^	B	11.4	T20/NVP/FTC/TDF
8		□	▪	□			□			□	□	10F, 20R, 36I, 36L, 46I, 62V, 63P, 71T, 90M, 93L	3^a^	B	4.5	ETR/TDF
9							▪		▪			36I, 46I, 63P, 69K, 82I, 89M, 93L	2^a^	C	26.6	FTC/TDF
10		▪	▪						▪	□		10I, 20T, 36I, 46I, 54V, 58E, 63P, 69K, 82L, 89M	6^a^	G	2.8	ddI/TDF
11	▪	▪	□				▪	□		□		10I, 36L, 43T, 46L, 54V, 58E, 63P, 82T, 89I	5	B	0.4^c^	T20/ETR/3TC/TDF

RAL, raltegravir; ABC, abacavir; 3TC, lamivudine; TDF, tenofovir; FTC, emtricitabine; T20, enfuvirtide; NVP, nevirapine; ETR, etravirine; ddI, didanosine; ▪, emergent mutation; □, baseline mutation.

^a^Participant received another PI for <90 days following baseline test.

^b^Time from start of darunavir to post-exposure genotype with emergent darunavir DRM.

^c^32I detected at 0.4 months, 11I and 54L detected at 9 months.

The selection pressure analysis included the 108 PI-naive study participants with subtype B infection and 216 controls who had received an NNRTI-containing regimen. The time between baseline and post-exposure genotypes was 18.7 months (IQR 6.7–35.6) for the PI-naive darunavir group and 18.7 months (6.8–36.4) for the NNRTI controls. Three protease positions were positively selected in at least two algorithms, of which two were not deemed to be a result of darunavir selection pressure as positive selection was also demonstrated in the NNRTI control group (protease positions 12 and 37); see Table 3. Codon 77 was the only site that was positively selected in the PI-naive group alone (FUBAR posterior probability = 0.99; FEL *P *= 0.017; SLAC *P *= 0.002).

**Table 3.** Protease gene positive selection analysis

Codon	PI-naive			Positive selection^d^	NNRTI controls			Positive selection^d^
	SLAC^a^	FEL^b^	FUBAR^c^		SLAC^a^	FEL^b^	FUBAR^c^	
12	**29.47 (0.004)**	∞ **(0.018)**	**0.18** **(0.96)**	+	**42.57** **(0.002)**	**4.90** **(0.045)**	0.10 (0.91)	+
35	17.96 (0.164)	2.48 (0.217)	0.12 (0.86)		**65.60** **(0.002)**	**4.56** **(0.018)**	**0.19** **(0.97)**	+
37	**75.10** **(<0.001)**	∞ **(<0.001)**	**0.649** **(1.0)**	+	**123.97** **(<0.001)**	2.08 (0.125)	**0.484** **(0.97)**	+
62	12.37 (0.269)	1.78 (0.551)	0.07 (0.73)		**59.36** **(0.002)**	∞ **(0.005)**	**0.221** **(0.98)**	+
63	0.28 (0.553)	1.22 (0.484)	0.02 (0.04)		37.51 (0.135)	**1.76** **(0.027)**	**0.435** **(0.99)**	+
64	**18.24** **(0.01)**	∞ (0.127)	0.10 (0.83)		10.11 (0.436)	0.84 (0.730)	−0.02 (0.48)	
77	**43.22** **(0.002)**	**6.50** **(0.017)**	**0.584** **(0.99)**	+	**43.59** **(0.018)**	2.07 (0.150)	0.11 (0.83)	
93	5.47 (0.437)	0.70 (0.541)	−0.15 (0.31)		**40.00** **(0.01)**	**3.61** **(0.044)**	0.16 (0.94)	+

^a^*dN* − *dS* (*P* value); *P *< 0.05 level of significance (the probability of observing as many or fewer synonymous changes, computed using an extended binomial distribution). *dN* is the non-synonymous substitution rate at the site. *dS* is the synonymous substitution rate at the site.

^b^*dN*/*dS* (*P* value); *P *< 0.05 level of significance using a likelihood test (*P* value of *dS *= *dN* versus *dS* ≠ *dN* test).

^c^β − α (posterior probability); posterior probability > 0.95 level of significance (probability α < β; the posterior probability of positive diversifying selection estimated by an empirical Bayes method). α is the posterior mean synonymous substitution rate. β is the posterior mean non-synonymous substitution rate.

^d^Site positively selected by at least two algorithms (bold text).

## Discussion

This national cohort study shows that <3% of people with baseline and post-darunavir genotypic tests developed emergent mutations, which supports the perception that darunavir has a high genetic barrier to resistance. PI-experienced participants were more likely to harbour darunavir DRMs at baseline than PI-naive individuals, probably as a result of cross-resistance from previously acquired protease mutations. None of the IAS-USA darunavir-associated mutations is unique to that agent and some people had been exposed to up to six different PIs before commencing darunavir. The PI-experienced group also had a greater incidence of emergent mutations than PI-naive participants. Possible explanations include the reappearance of archived mutations that had been selected previously by other PI agents, but were not replicating during baseline testing. Alternatively, there may have been resistant minority variants circulating at levels too low to detect by standard Sanger sequencing, which then flourished under the selective pressure of darunavir therapy to become the consensus viral sequence at that protease position. This hypothesis is supported by the rapid emergence of V32I after only 13 days of therapy in a person who had been treated previously with five different PIs, which suggests the presence of existing DRMs within the viral population rather than a spontaneous occurrence. In the PI-naive group it is more likely that emergent resistance mutations arose *de novo* and then replicated during therapy.

The selection analysis was used to explore the evolution of subtype B protease genes under the selective pressure of darunavir. This showed that non-synonymous mutations at codon 77 were positively selected during darunavir therapy, compared with matched PI-naive controls receiving an NNRTI-based regimen. Codon 77 is a polymorphic site that has been identified as one of the strongly positively selected sites in protease.^20^ In the Stanford HIV drug resistance database, 66% of subtype B protease sequences from PI-naive patients code for valine (V) at this position and 34% for isoleucine (I).^21^ The V77I substitution is associated with minor resistance to older PIs, namely indinavir, saquinavir and nelfinavir, but has not been implicated in darunavir resistance. The adjacent position, 76, is a known resistance-associated site so it may be that mutations of codon 77 represent compensatory changes that are important for the development of darunavir resistance. An *in silico* structural study has shown that HIV-1 proteases containing the V77I substitution impact the cavity size of the protease active site and its binding affinity for nelfinavir.^22^ Phenotypic experiments may be warranted to explore whether codon 77 is involved in darunavir susceptibility. It is surprising that the present analysis did not identify more positively selected darunavir resistance-associated sites, but this could reflect the rarity of emergent resistance to this agent.

This analysis of data from clinical practice settings indicates that emergent darunavir resistance is uncommon. This corroborates the findings of clinical trials, including the TITAN study, in which only 1.7% (5/298) of patients in the darunavir arm developed emergent darunavir DRMs not detected in previous testing, all of whom were PI-experienced prior to enrolment.^8^ This compared with 6.4% (19/297) of patients who developed emergent lopinavir DRMs in the lopinavir arm. The most common emergent darunavir mutations were V32I, L33F, I47V, I54L, T74P and L76V.^23^ In the ARTEMIS trial, 343 treatment-naive participants were randomized to a darunavir-containing regimen and there were 4 cases of emergent minor PI mutations in the 43 participants with baseline and repeat genotypes.^9^ The ODIN trial compared once-daily darunavir with twice-daily dosing in addition to an optimized background regimen in 590 treatment-experienced patients.^24^ Around half were PI-experienced and those with baseline darunavir DRMs were excluded. Baseline and endpoint genotypes were available for 102 patients, but only 2 developed emergent darunavir mutations (one participant in the once-daily arm developed V32I, M46I, L76V and I84V and another in the twice-daily arm developed L33F and I50V).^23^ Emergent resistance was seen more frequently in trials with highly treatment-experienced patients. A pooled analysis of the POWER studies and the darunavir arms of the DUET trials, which compared darunavir with etravirine, found that 41% of participants with virological failure had developed V32I and 25% developed I54L or I54M. Other mutations that emerged during these trials were V11I, I15V, L33F, I47V, I50V and L89V.^23^

There are limited data on the protease mutations that emerge during darunavir therapy in other clinical practice settings. A French study described 25 highly treatment-experienced patients, with an average of five previous PIs, who failed darunavir therapy.^25^ The median time from darunavir initiation to sampling was 34 weeks (IQR 12–104), by which point 18 patients had developed darunavir DRMs including V11I, V32I, L33F/I, I47V/A, I50V, I54L/M and L89I/M/V. A greater risk of emergent mutations was seen in those with two or three baseline DRMs and with ongoing viral replication after 24 weeks of treatment. A Spanish cohort of 24 patients who failed darunavir salvage therapy each developed a median of three emergent darunavir DRMs (IQR 1–4), including V11I, V32I, L33F, I47V, I54L, L54M, I84V and L89V.^26^ There is considerable overlap in the mutations that emerged in clinical trials and cohort studies and those observed in the present analysis (V11I, V32I, L33F, I54L, L76V and I84V). The previous cohort studies reported a much higher risk of emergent resistance than we observed; however, the inclusion of patients only receiving darunavir as a last resort, following multiple treatment regimen failures, limits their external validity to the broader clinical setting in the UK, where darunavir is widely used as first- and second-line therapy. A 2011 analysis of the UKHDRD looked at the response to lopinavir-containing regimens in PI-naive participants.^27^ Eight hundred and eleven participants experienced virological failure and 286 had a resistance test following failure. Of those, 32 (11%) had protease resistance mutations and the most common were I54V, M46I, V82A and L76V. This study showed a higher prevalence of lopinavir-associated DRMs during failure on this agent than we found of darunavir-associated DRMs during darunavir in the same population. It may be expected that darunavir would perform better in this regard; however, the two studies are not directly comparable. The lopinavir study participants did not have baseline resistance tests for comparison, and the fact that this analysis was conducted several years ago means that there could be differences in clinical practice in terms of when a PI may be prescribed and requesting resistance tests during virological failure.

A strength of the present study is the large number of study participants, receiving care at a range of HIV treatment centres, ensuring a sample that is broadly representative of the darunavir-treated population in the UK. Another advantage is that, in contrast to most studies of genotypic resistance, this analysis examines the within-host evolution of the protease gene during darunavir therapy rather than relying on the baseline sequence alone to infer genetic predictors of failure. However, this approach also has some limitations. For example, emergent mutations could have been missed if clinicians were less likely to request resistance testing following virological failure with darunavir because they did not expect this agent to cause significant resistance. Conversely, the rate of emergent resistance attributable to darunavir could have been overestimated in this study as five patients with emergent DRMs had received other PIs following baseline testing. A further drawback is the lack of information on darunavir dosing frequency. A higher dose of darunavir (600 mg twice daily, instead of the usual 800 mg once daily) tends to be used in patients with baseline darunavir-associated mutations and this practice may impact on the development of emergent resistance. Another shortcoming is that the HIV *pol* sequencing routinely performed for clinical care uses the Sanger methodology, which can only reliably detect minority species down to ∼20% prevalence. It is hoped that next-generation sequencing technology will enable a fuller depiction of viral population dynamics and quantification of resistant minority variants. Longitudinal studies using this technology could explore the role of pre-existing minority variants in emerging resistance. WGS may also reveal mutations in regions other than *pol*, such as *gag*, which has been implicated in the development of PI resistance.^28–31^ This study confirms that emergent darunavir resistance is rare in UK clinical settings. However, multiple darunavir mutations do emerge during therapy in a minority of patients, and therefore repeat genotyping in the case of poor virological response may still be warranted to detect resistance and guide management decisions.

## 

### UK HIV Drug Resistance Database

#### Steering Committee

Celia Aitken (Gartnavel General Hospital, Glasgow); David Asboe, Anton Pozniak (Chelsea & Westminster Hospital, London); Patricia Cane (Public Health England, Porton Down); David Chadwick (South Tees Hospitals NHS Trust, Middlesbrough); Duncan Churchill (Brighton and Sussex University Hospitals NHS Trust); Duncan Clark (St Bartholomew's and The London NHS Trust); Simon Collins (HIV i-Base, London); Valerie Delpech (Centre for Infections, Public Health England); Samuel Douthwaite (Guy's and St Thomas' NHS Foundation Trust, London); David Dunn, Esther Fearnhill, Kholoud Porter, Anna Tostevin, Ellen White (MRC Clinical Trials Unit at UCL, London); Christophe Fraser (Imperial College London); Anna Maria Geretti (Institute of Infection and Global Health, University of Liverpool); Antony Hale (Leeds Teaching Hospitals NHS Trust); Stéphane Hué (London School of Hygiene and Tropical Medicine, London); Steve Kaye (Imperial College, London); Paul Kellam (Wellcome Trust Sanger Institute & University College London Medical School); Linda Lazarus (Expert Advisory Group on AIDS Secretariat, Public Health England); Andrew Leigh-Brown (University of Edinburgh); Tamyo Mbisa (Virus Reference Department, Public Health England); Nicola Mackie (Imperial NHS Trust, London); Samuel Moses (King's College Hospital, London); Chloe Orkin (St Bartholomew's Hospital, London); Eleni Nastouli, Deenan Pillay, Andrew Phillips, Caroline Sabin (University College London Medical School, London); Erasmus Smit (Public Health England, Birmingham Heartlands Hospital); Kate Templeton (Royal Infirmary of Edinburgh); Peter Tilston (Manchester Royal Infirmary); Ian Williams (Mortimer Market Centre, London); Hongyi Zhang (Addenbrooke's Hospital, Cambridge).

#### Coordinating centre

MRC Clinical Trials Unit at UCL (David Dunn, Keith Fairbrother, Esther Fearnhill, Kholoud Porter, Anna Tostevin, Ellen White)

#### Centres contributing data

Clinical Microbiology and Public Health Laboratory, Addenbrooke's Hospital, Cambridge (Jane Greatorex); Guy's and St. Thomas' NHS Foundation Trust, London (Siobhan O'Shea, Jane Mullen); PHE—Public Health Laboratory, Birmingham Heartlands Hospital, Birmingham (Erasmus Smit); PHE—Virus Reference Department, London (Tamyo Mbisa); Imperial College Health NHS Trust, London (Alison Cox); King's College Hospital, London (Richard Tandy); Medical Microbiology Laboratory, Leeds Teaching Hospitals NHS Trust (Tracy Fawcett); Specialist Virology Centre, Liverpool (Mark Hopkins, Lynne Ashton); Department of Clinical Virology, Manchester Royal Infirmary, Manchester (Peter Tilston); Department of Virology, Royal Free Hospital, London (Clare Booth, Ana Garcia-Diaz); Edinburgh Specialist Virology Centre, Royal Infirmary of Edinburgh (Jill Shepherd); Department of Infection & Tropical Medicine, Royal Victoria Infirmary, Newcastle (Matthias L. Schmid, Brendan Payne); South Tees Hospitals NHS Trust, Middlesbrough (David Chadwick); Department of Virology, St Bartholomew's and The London NHS Trust (Duncan Clark, Jonathan Hubb); Molecular Diagnostic Unit, Imperial College, London (Steve Kaye); University College London Hospitals (Stuart Kirk); West of Scotland Specialist Virology Laboratory, Gartnavel, Glasgow (Rory Gunson, Amanda Bradley-Stewart, Celia Aitken).

### UK CHIC Study

#### Steering Committee

Jonathan Ainsworth (North Middlesex University Hospital NHS Trust, London), Sris Allan (University Hospitals Coventry and Warwickshire NHS Trust), Jane Anderson (Homerton University Hospital NHS Trust, London), Abdel Babiker (MRC Clinical Trials Unit, London), David Chadwick (South Tees Hospitals NHS Trust, Middlesbrough), Valerie Delpech, PHE, London), David Dunn (MRC Clinical Trials Unit, London), Martin Fisher (Brighton and Sussex University Hospitals NHS Trust), Brian Gazzard (Chelsea and Westminster Hospital NHS Foundation Trust, London), Richard Gilson (Mortimer Market Centre, Central and North West London NHS Foundation Trust), Mark Gompels (North Bristol NHS Trust), Phillip Hay (St George's Healthcare NHS Trust), Teresa Hill (University College London), Margaret Johnson (Royal Free Foundation NHS Trust, London), Sophie Jose (University College London), Stephen Kegg (Lewisham and Greenwich NHS Trust, London), Clifford Leen (Lothian University Hospitals NHS Trust), Nicola Mackie (Imperial College Healthcare NHS Trust, London), Fabiola Martin (York Teaching Hospital NHS Foundation Trust), Mark Nelson (Chelsea and Westminster Hospital NHS Trust, London), Chloe Orkin (Barts Health NHS Trust, London), Adrian Palfreeman (University Hospitals of Leicester NHS Trust), Andrew Phillips (University College London), Deenan Pillay (University College London), Frank Post (King's College Hospital NHS Foundation Trust, London), Jillian Pritchard (Ashford and St Peter's Hospitals NHS Foundation Trust), Caroline Sabin (University College London), Memory Sachikonye (UK Community Advisory Board), Achim Schwenk (North Middlesex University Hospital NHS Trust, London), Anjum Tariq (The Royal Wolverhampton Hospitals NHS Trust), John Walsh (Imperial College Healthcare NHS Trust, London).

#### Central coordination

University College London (Teresa Hill, Sophie Jose, Andrew Phillips, Caroline Sabin, Alicia Thornton); Medical Research Council Clinical Trials Unit at UCL (MRC CTU at UCL), London (David Dunn, Adam Glabay).

#### Participating centres

Barts Health NHS Trust, London (Chloe Orkin, Janet Lynch, James Hand, Carl de Souza); Brighton and Sussex University Hospitals NHS Trust (Martin Fisher, Nicky Perry, Stuart Tilbury, Elaney Youssef, Duncan Churchill); Chelsea and Westminster Hospital NHS Foundation Trust, London (Brian Gazzard, Mark Nelson, Rhiannon Everett, David Asboe, Sundhiya Mandalia); PHE, London (Valerie Delpech); Homerton University Hospital NHS Trust, London (Jane Anderson, Sajid Munshi); King's College Hospital NHS Foundation Trust, London (Frank Post, Ade Adefisan, Chris Taylor, Zachary Gleisner, Fowzia Ibrahim, Lucy Campbell); MRC CTU, London (Abdel Babiker, David Dunn, Adam Glabay); Middlesbrough, South Tees Hospitals NHS Foundation Trust, (David Chadwick, Kirsty Baillie); Mortimer Market Centre, University College London (Richard Gilson, Nataliya Brima, Ian Williams); North Middlesex University Hospital NHS Trust, London (Jonathan Ainsworth, Achim Schwenk, Sheila Miller, Chris Wood); Royal Free NHS Foundation Trust/University College London (Margaret Johnson, Mike Youle, Fiona Lampe, Colette Smith, Rob Tsintas, Clinton Chaloner, Samantha Hutchinson, Caroline Sabin, Andrew Phillips, Teresa Hill, Sophie Jose, Alicia Thornton, Susie Huntington); Imperial College Healthcare NHS Trust, London (John Walsh, Nicky Mackie, Alan Winston, Jonathan Weber, Farhan Ramzan, Mark Carder); The Lothian University Hospitals NHS Trust, Edinburgh (Clifford Leen, Alan Wilson, Sheila Morris); North Bristol NHS Trust (Mark Gompels, Sue Allan); Leicester, University Hospitals of Leicester NHS Trust (Adrian Palfreeman, Khurram Memon, Adam Lewszuk); Woolwich, Lewisham and Greenwich NHS Trust (Stephen Kegg, Akin Faleye, Sue Mitchell, Meg Hunter), UK Community Advisory Board (Memory Sachikonye); St George's Healthcare NHS Trust (Phillip Hay, Mandip Dhillon, Christian Kemble); York Teaching Hospital NHS Foundation Trust (Fabiola Martin, Sarah Russell-Sharpe, Janet Gravely); Coventry, University Hospitals Coventry and Warwickshire NHS Trust (Sris Allan, Andrew Harte, Stephen Clay); Wolverhampton, The Royal Wolverhampton Hospitals NHS Trust (Anjum Tariq, Hazel Spencer, Ron Jones); Chertsey, Ashford and St Peter's Hospitals NHS Foundation Trust (Jillian Pritchard, Shirley Cumming, Claire Atkinson).

## Funding

This work was supported by the UK Medical Research Council (Award number 164587). K. E. B. is funded by the Wellcome Trust (Award number 170461).

## Transparency declarations

C. A. S. has received honoraria for participation in Data Safety and Monitoring Boards, Advisory Boards and Speaker Panels and for the preparation of educational material from Gilead Sciences, ViiV Healthcare, Janssen-Cilag and MSD. A. N. P. has in the past 3 years received payment for consultancy (Gilead Sciences and GSK Biologicals), speaking (Gilead Sciences) and participation in Advisory Boards (AbbVie). N. M. has received honoraria for participation in Advisory Boards and Speaker Panels and for the preparation of educational material from ViiV Healthcare and Gilead Sciences. A. L. P. has received honoraria for participation in Speaker Symposia and Advisory Boards from Merck, ViiV Healthcare, Gilead Sciences, BMS and Janssen-Cilag. All other authors: none to declare.
